# Polymersome Poration and Rupture Mediated by Plasmonic Nanoparticles in Response to Single-Pulse Irradiation

**DOI:** 10.3390/polym12102381

**Published:** 2020-10-16

**Authors:** Gina M. DiSalvo, Abby R. Robinson, Mohamed S. Aly, Eric R. Hoglund, Sean M. O’Malley, Julianne C. Griepenburg

**Affiliations:** 1 Department of Chemistry, Rutgers University-Camden, 315 Penn Street, Camden, NJ 08102, USA; gdisalvo22@gmail.com (G.M.D.); robinsonabby13@gmail.com (A.R.R.); 2 Department of Physics, Rutgers University-Camden, 227 Penn Street, Camden, NJ 08102, USA; msa194@scarletmail.rutgers.edu (M.S.A.); omallese@camden.rutgers.edu (S.M.O.); 3 Department of Materials Science and Engineering, University of Virginia, Thornton Hall, P.O. Box 400259, Charlottesville, VA 22904, USA; erh3cq@virginia.edu; 4 Center for Computational and Integrative Biology, Rutgers University-Camden, Camden, NJ 08102, USA

**Keywords:** polymersomes, vesicles, nanoparticles, drug-delivery, ultrafast laser, plasmonic, nanobubble, fragmentation

## Abstract

The self-assembly of amphiphilic diblock copolymers into polymeric vesicles, commonly known as polymersomes, results in a versatile system for a variety of applications including drug delivery and microreactors. In this study, we show that the incorporation of hydrophobic plasmonic nanoparticles within the polymersome membrane facilitates light-stimulated release of vesicle encapsulants. This work seeks to achieve tunable, triggered release with non-invasive, spatiotemporal control using single-pulse irradiation. Gold nanoparticles (AuNPs) are incorporated as photosensitizers into the hydrophobic membrane of micron-scale polymersomes and the cargo release profile is controlled by varying the pulse energy and nanoparticle concentration. We have demonstrated the ability to achieve immediate vesicle rupture as well as vesicle poration resulting in temporal cargo diffusion. Additionally, changing the pulse duration, from femtosecond to nanosecond, provides mechanistic insight into the photothermal and photomechanical contributors that govern membrane disruption in this polymer–nanoparticle hybrid system.

## 1. Introduction

The ability to deliver cargo from carrier vesicles with high spatiotemporal control would greatly enhance a variety of applications, including nanoreactors, microreactors, and drug delivery systems. Liposomes have been an extensively investigated carrier system for targeted release and their implementation in nanomedicine has resulted in significant advances in therapeutic compound stability, tissue uptake, and biodistribution in vivo [1]. However, many challenges exist with liposome systems such as long-term stability and membrane permeability. To overcome these limitations, attention has been redirected to their fully synthetic polymeric analogs, known as “polymersomes” [2].

Polymersomes are a class of fully synthetic vesicles which self-assembly from a variety of amphiphilic diblock copolymers [3]. The self-assembly process results in two distinct regions: a bilayer member that contains a hydrophobic center, and an aqueous core which can hold hydrophilic encapsulants. The properties of the bilayer membrane are readily tunable by adjusting the molecular weights (MW) of the hydrophobic and hydrophilic blocks of the amphiphile. For example, a hyperthick bilayer membrane up to 50 nm, allowing for storage of hydrophobic encapsulants, can be achieved by using high MW amphiphiles [4]. This robust, dual-compartment encapsulation ability makes polymersomes an ideal candidate for applications which require molecular transport in an unreacted state. For these systems to garner success in application, the ability to release cargo with high spatiotemporal control is of utmost importance. The design of stimuli responsive polymersomes has been extensively investigated, as summarized by Che et al., resulting in the development of polymersomes that are responsive to a variety of external stimuli (e.g., ultrasound, magnetic fields, light) or internal stimuli (e.g., temperature, pH, redox) [5].

Light is a particularly attractive trigger as it is minimally damaging yet deeply penetrating at certain wavelengths (and intensities). It can also be localized in time and space, and externally triggered without the need for additional reagents [5,6]. While there exist several examples of light stimulated encapsulant release from polymersomes, many require the use of high-energy (UV) light to trigger cleavable groups such as nitrobenzyl or azobenzene [7,8,9,10]. Examples of polymersomes that rupture in response to light in the visible to near-infrared (NIR) window involve the use of conjugated multi-porphyrin dyes encapsulated within the hydrophobic membrane [11]. Kamat et al. and Griepenburg et al. show vesicle deformation and rupture of micron- and nano-scale vesicles, respectively; however, this is achieved with long irradiation times using combined, continuous wave (CW) light at multiple wavelengths across the visible region [12,13,14].

Plasmonic nanoparticles are ideal candidates as photosensitizers to initiate light-triggered release due to the photothermal response accompanying excitation of their localized surface plasmon resonance (LSPR) and large optical cross sections. One particularly notable example includes the work by Amstad et al. where hydrophobic gold nanoparticles (NPs) (AuNPs) were incorporated into the membrane of polymersomes self-assembled from poly(ethylene glycol)-b-poly(lactic acid) (PEG-b-PLA) and poly(N-isopropylacrylamide)-b-poly(lactic-co-glycolic acid) (PNIPAM-b-PLGA) diblock copolymers [15]. Vesicles were irradiated with three continuous wave (CW) lasers simultaneously (488, 532, and 633 nm) resulting in a decrease in intact vesicles. Under irradiation, the thermoresponsive PNIPAM blocks underwent amphiphilicity changes in response to local hot spots generated by the AuNPs, ultimately resulting in complete destabilization of the bilayer. Additional work by Zhang et al. includes the use of porous silicon nanoparticles conjugated with gold nanorods that are encapsulated within the aqueous core of hybrid poly(ethylene glycol)-b-poly(lactic acid) nanopolymersomes to achieve hydrophobic and hydrophilic drug loading capacities as well as photothermal responsiveness. These nanovesicles were shown to suppress breast tumors in mice by up to 94% through loading with a triple-drug combination [16].

One drawback common to the aforementioned systems is the need for long irradiation times, which is inherent to the use of CW irradiation. Such long irradiation times are not ideal in dynamic systems as this can result in decreased localization of the released encapsulant. In contrast, pulsed, and in particular, ultrafast laser irradiation, holds great promise for the use in delivery applications, due primarily to the ability of achieving high peak power in short durations, thus allowing for a photomechanical response which would enhance localization.

Several notable examples exist for using nanoparticle excitation to trigger destabilization of liposomes and subsequent cargo release; however, only a few utilize pulsed laser systems [17,18]. In a closely related example, Mathiyazhakan et al. highlight a system where hydrophilic AuNPs are loaded into the core of a thermo-responsive liposome [19]. The ability to release a model drug, calcein, was demonstrated with pulsed laser irradiation, and the mechanism through which membrane disruption occurred was attributed to microbubble cavitation. This example, however, required several nanosecond pulses (25–100) to achieve a significant percentage of release. The authors acknowledge that CW irradiation failed to trigger release from their system. Wu et al. studied cargo release from liposomes with hollow gold nanoshells incorporated at various locations (tethered, freely outside, inside the aqueous core) under ultrafast irradiation, and determined that membrane–NP proximity is an important factor in release profiles [20]. These examples reinforce the benefits of polymersomes, where nanoparticles can more readily be incorporated into their relatively thick bilayer membrane, facilitating closer proximity, and thus more efficient release without the need for thermoresponsive diblock copolymers.

**Figure 1:**
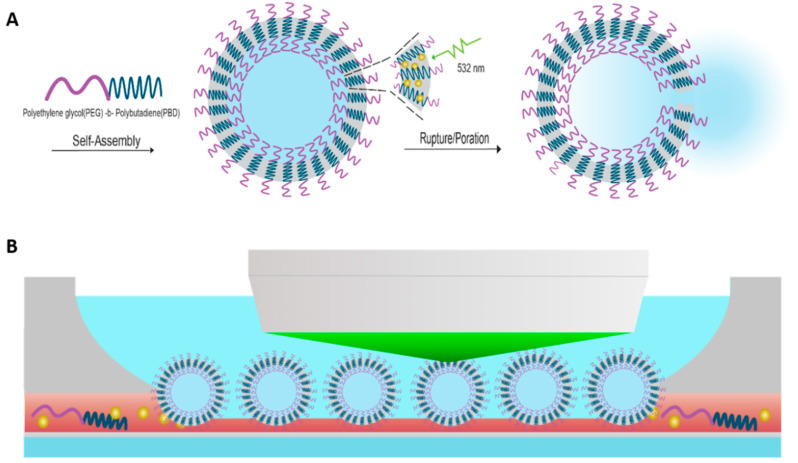
(**A**) Schematic representation of hydrophobic, 2.5 nm, plasmonic gold nanoparticles (AuNPs) incorporated into the hydrophobic membrane of micron-scale polyethylene oxide-*b*-polybutadiene (PBD_35_-b-PEO_20_) polymersomes during the self-assembly process. Single-pulse irradiation on resonance with the localized surface plasmon resonance (LSPR) of the AuNPs results in polymersome rupture or poration. (**B**) Irradiation studies are performed at the single-vesicle level, by focusing the laser through an optical objective which allows for irradiation and subsequent imaging.

Herein, as shown in Figure 1, we demonstrate the use of 2.5 nm, hydrophobic, plasmonic gold nanoparticles (AuNP) incorporated into the hydrophobic membrane of micron-scale polymersomes as efficient photosensitizers for polymersome poration and/or rupture. The use of pulsed lasers is chosen to decrease irradiation time, and thereby potentially increase temporal resolution over conventional CW systems. The diblock copolymer poly(butadiene)-b-poly(ethylene oxide) PBD_35_-b-PEO_20_ was chosen given that it is robust and not inherently photoresponsive or thermoresponsive in temperature ranges of interest for this study as well as future applications in vitro and in vivo [21]. Single-vesicle imaging studies demonstrate that encapsulant release from polymersomes can be effectively controlled via pulse energy and AuNP concentration. Additionally, the mechanisms by which membrane disruptions are brought about are investigated through laser pulse duration studies.

## 2. Materials and Methods

### 2.1. Micron-Scale Polymersome Preparation

Polymersomes were prepared from the diblock copolymer, polyethylene oxide-*b*-polybutadiene (PBD_35_-*b*-PEO_20_) (Polymer Source, Montreal, QC, Canada) by the gel-assisted rehydration method [22]. The diblock copolymer was dissolved in chloroform at a concentration of 5 mg/mL. To visualize the vesicles under fluorescence microscopy, Nile red (Santa Cruz Biotechnology, Dallas, TX, USA) was added to the organic copolymer solution to yield a final concentration of 0.5 mol%. This step also includes the addition of 2–5 nm dodecanethiol functionalized spherical AuNPs (Alfa Aesar, Haverhill, MA, USA) to the organic solution, in concentrations varying from 0.013–0.066% (*w/v*). The organic solution was spread atop an agarose coated coverslip. The agarose coated coverslips were prepared by spreading a 1% (*w/v*) agarose solution (Sigma Aldrich, St. Louis, MO, USA) atop a 25 × 50 × 0.13 − 0.17 mm glass coverslip (Electron Microscopy Sciences, Hatfield, PA, USA) using a 1000 µL pipette tip. The agarose coated coverslips were then dried at 37 °C in a vacuum oven to fully remove the solvent, resulting in a thin agarose film. Organic solution (55 µL) containing copolymer, nanoparticle, and Nile red fluorophore was spread on the agarose coated coverslip with the side of a 12-gauge needle until the film visibly dried. The polymer coated coverslip was then further dried under vacuum for at least 1 h. A custom-made polydimethylsiloxane (PDMS) well, prepared from a Dow SYLGARDTM 170 Silicone Encapsulant Kit (Ellsworth Adhesives, Germantown, WI, USA), was adhered to the top of the polymer and agarose coated coverslip. This allows for the rehydration buffer to be added and contained on top of the polymer coated agarose coverslip. The rehydration buffer (600 µL) was added to the well. At this step, any hydrophilic components to be encapsulated within the polymersome core were added to the rehydration buffer. The hydrophilic fluorophore, 3–5k molecular weight fluorescein isothiocyanate-dextran (FITC-dextran) (Sigma Aldrich, St. Louis, MO, USA) was added to the 280 mM sucrose (Sigma Aldrich, MO, USA) rehydration buffer to a final concentration of 0.5 mg/mL. Upon addition of the rehydration buffer and heating ranging from 45 °C–65 °C for 45 min, polymersomes form and remain partially attached to the agarose gel. Figure S1 shows a schematic representation of the gel rehydration procedure. Vesicles prepared via this protocol had a mean diameter of 30.3 µm with a standard deviation of 8.5 µm. During the irradiation experiments, vesicles remain attached to the coverslip with the hydration well in place, to ensure that vesicles are static and in an aqueous environment.

### 2.2. Polymersome Imaging

Upon polymersome formation, the sample was imaged by fluorescence microscopy using a ZEISS Axio Examiner, fixed stage upright microscope, equipped with a 20× (0.5 numerical aperture) dipping objective which aided in visualization of the vesicles located at the bottom of the rehydration well, as depicted in Figure 1B. During the irradiation step, the fluorescent cube was rotated out of position to allow the beam to pass through to the objective unobstructed, and then moved back into position to allow for post-irradiation monitoring of the vesicle and its fluorescent encapsulants.

### 2.3. Removal of Excess Encapsulant

**Figure 2:**
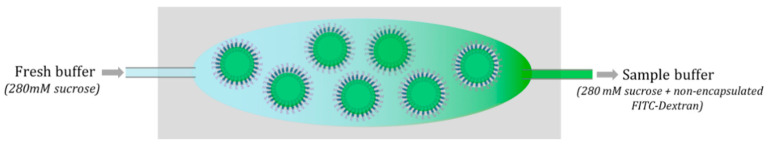
Top-down schematic for buffer exchange procedure. Buffer exchange is required for removal of non-encapsulated aqueous molecules. Fluorescein isothiocyanate-dextran (FITC-dextran) is included in the hydration buffer for encapsulation within the polymersome core. Excess FITC-dextran which remains in the buffer must be removed in order to facilitate imaging of the polymersome core.

For polymersomes prepared with encapsulants, e.g., fluorescein isothiocyanate-dextran (FITC-dextran), within the vesicle core, a buffer exchange was necessary to rid the sample of the non-encapsulated components. This helped reduce the background fluorescence and facilitated imaging of the vesicle core. Two pieces of tubing (0.012 “IDx0.030” OD, Cole Parmer, Vernon Hills, IL, USA) were inserted into the buffer solution through the side walls of the custom PDMS well. Fresh buffer was slowly pumped into the well from one syringe pump, while the sample buffer containing free FITC-dextran was simultaneously pulled from the well using a second syringe pump (Figure 2). A gentle flow prevents vesicles from lifting off of the agarose gel.

### 2.4. Pulsed Laser Irradiation

Irradiation experiments were made feasible through a unique multi-functional optical microscope integrated with femtosecond and nanosecond laser systems. The ultrafast system is a Spectra Physics Solstice Ti:sapphire (100 fs) pumping a Light Continuum optical parametric amplifier (OPA) with the output set to 532 nm. The nanosecond laser is a Ekspla NL201 (7 ns) diode pumped Nd:YAG system equipped with a second harmonic generation crystal to an output wavelength of 532 nm. The beams are passed through a pair of filter wheels, to adjust attenuation, before being steered into an auxiliary port located above the filter carousel of the microscope and made collinear with the optical path via a dichroic mirror. This configuration, as demonstrated in Figure 1B and pictured in Figure S2, allows for irradiation and subsequent imaging of micron-sized vesicles. For micron-sized polymersomes, individual vesicles were singled-out and irradiated with a single pulse and subsequently imaged through a time-series to monitor vesicle integrity and encapsulant release. All imaging and irradiation experiments were carried out at 21 °C.

The laser spot size was measured and adjusted through the 20× dipping objective to ensure the laser beam was focused directly on the polymersome to be irradiated. This spot size was measured with a damage spot on a glass bottom petri dish where applied ink spots were submerged in water and irradiated as shown in Figure S3. The pulse energy was attenuated and measured using an energy meter placed on the microscope stage at the location of sample irradiation. No objective was used for this measurement, however, an associated 8% reduction in energy was taken into account when reporting pulse energy.

Zeiss ZenPro 2.5 software was utilized for analysis of the polymersome time series following irradiation. To quantify release, a region of interest (ROI) was selected inside of the polymersome as well as a background region to normalize the signal and account for photobleaching. A sample data series is shown in Table S1.

### 2.5. Studying the Fate of the Incorporated AuNPs upon Polymersome Irradiation

Polymersome samples containing 0.05% (*w/v*) AuNPs were prepared as described. Nano-polymersomes were self-assembled as described below and the sample was pipetted into a PDMS well placed atop a glass slide, to which a small magnetic stir bar was added. The sample was then placed under the dipping objective of the upright microscope and continuously irradiated at 532 nm from either the femtosecond system (80 nJ, 100 fs, 500 Hz) for 20 min or the nanosecond laser (10.3 µJ, 7 ns, 500 Hz) for 20 min. Gold nanoparticle size distributions were determined by high-resolution transmission electron microscopy using a 300 kV FEI Titan system equipped with a Gatan 794 camera, achieving a maximum resolution of 0.205 nm. Nanoparticle size distributions were determined by measuring the feret diameter for a minimum of 5000 particles across multiple images per sample using ImageJ. Representative images have been included in Figure S4.

### 2.6. Nano-Scale Polymersome Preparation

Nano-scale vesicles were prepared via the direct solvent injection method. The diblock copolymer, PEO_20_-*b*-PBD_35_ (Polymer Source, Montreal, QC, Canada), was dissolved in tetrahydrofuran (THF) (Sigma Aldrich, St. Louis, MO, USA) to yield a concentration of 4 mg/mL, unless otherwise stated. Incorporation of various concentrations of AuNPs to the polymer-THF solution took place by a resuspension process where 2–5 nm dodecanethiol functionalized, spherical AuNPs (Alpha Aesar, Haverhill, MA, USA) suspended in toluene (2% *v*/*v*), were transferred to an open Eppendorf tube and gently heated to aid in solvent evaporation. The dry AuNPs were then re-suspended by adding 300 µL of the polymer–THF solution. To prepare for solvent injection, a stir bar was added to a glass vial containing 700 µL double deionized (DDI) (Milli-Q) water, and parafilm used to seal the vial. The vial containing the aqueous solution was subjected to magnetic stirring. The copolymer–AuNP–THF solution was drawn into a 3 mL syringe and a 23 gauge 1” blunt tip needle with tubing (0.012 “IDx0.030” OD) (Cole Parmer, Vernon Hills, IL, USA) was attached. The organic solution was added dropwise (10 µL/second) to the aqueous solution under continuous stirring, which created an emulsion resulting in the self-assembly of nano-polymersomes. The polymersome sample was then filtered using a 0.45 µm polytetrafluoroethylene (PTFE) syringe filter (GS-TEK, Newark, DE, USA) to remove any large aggregates which possibly formed from copolymer and/or AuNPs which did not assemble into vesicular structures.

### 2.7. Size Confirmation

Nano-polymersome size was determined by measuring the hydrodynamic diameter via dynamic light scattering (DLS) using a Malvern Zetasizer Nano ZS with a scattering angle set to 173°. The sample was filtered using a 0.45 µm syringe filter, as described above, to ensure that any large particulates (i.e., non-vesicular aggregates, dust) did not bias the measurement. The polymersome sample was diluted (~33-fold), using DDI H_2_O, in a polystyrene cuvette to a final volume of 1 mL and the sample was measured in triplicate, with each run consisting of a minimum of 10 scans at 25 °C. While cryogenic transmission electron microscopy (cryo-TEM) is often regarded as the gold-standard for size determination of nano-vesicles smaller than 200 nm, DLS can be used as a simple screening technique when accompanied with careful analysis [23,24]. Nano-polymersome formation was screened by confirming that both the intensity distribution and Z-Average size were between 50 and 100 nm and the polydispersity index (PDI) was used to determine uniformity for each sample. Non-irradiated, nanosecond (ns)-irradiated, and femtosecond (fs)-irradiated samples were determined to have average hydrodynamic diameters by intensity of 71 nm (98.8%), 67 nm (98.5%), and 75 nm (98.2%), respectively, all with a PDI of 0.200 or less. As these samples were relatively monodisperse, the Z-Average sizes, based on the harmonic mean calculated within the software (Zetasizer v7.11), correlate well with intensity distributions, at 61.2 nm, 60.4 nm, and 63.0 nm.

## 3. Results

### 3.1. AuNPs as Photosensitizers for Complete Polymersome Rupture

Initial studies sought to determine the sensitivity of micron-scale PEO_20_-*b*-PBD_35_ polymersomes to single-pulse, femtosecond laser irradiation, and whether the addition of AuNPs within the hydrophobic membrane would increase photosensitivity.

**Figure 3:**
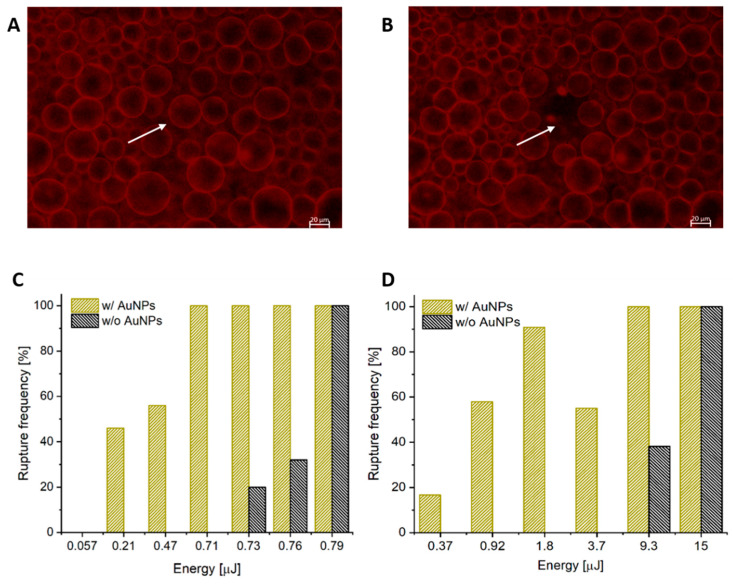
(**A**) Polymersomes with Nile red and 0.066% (*w/v*) AuNPs encapsulated in the hydrophobic region of the membrane, prior to laser irradiation. (**B**) The polymersome indicated by the white arrow was irradiated with a single 532 nm fs pulse and immediately imaged. Complete rupture of the polymersome is observed while the surrounding vesicles remain intact. Scale bar represents 20 µm. (**C**) Rupture statistics for polymersomes upon femtosecond irradiation. Rupture frequency of polymersomes with AuNPs within the membrane (gold) and without AuNPs (black) are shown. These percentages represent the number of vesicles undergoing complete rupture upon irradiation by a single 532 nm fs pulse at the specified energies. A minimum of 20 vesicles were irradiated per pulse energy. (**D**) Rupture statistics for polymersomes using nanosecond irradiation. The percentage of polymersomes rupturing in response to varying ns pulse energies was determined for populations of vesicles with (gold) and without (black) AuNPs in the membrane. These percentages represent complete rupture upon irradiation by a single 532 nm, ns pulse at the specified energies.

As shown in Figure 3, complete vesicle rupture is observed immediately following irradiation with a single pulse at 532 nm, while the surrounding, non-irradiated vesicles remain intact. As a control, polymersomes without AuNPs in the hydrophobic membrane were subjected to identical fs irradiation and it was determined through interpolation that 5× the energy was required to initiate rupture. It follows that complete vesicle rupture would result in immediate content release of hydrophilic encapsulants to the surrounding environment.

To further investigate the effect of AuNP encapsulation within the hydrophobic membrane of the polymersome, Figure 3C shows the rupture frequency as a function of pulse energy for both populations of vesicles with and without AuNPs. No fewer than 20 similarly sized vesicles were irradiated at each energy and only vesicles with no more than one contact point with another vesicle were chosen for this study. Vesicles selected for irradiation had an average diameter of 45.2 ± 6.8 µm. As shown in Figure 3C, pulse energy required to induce observable rupture for vesicles containing AuNPs with fs-irradiation was 0.21 µJ, thus indicating a rupture threshold in the 0.057–0.21 µJ range. The energy required to observe rupture in vesicles w/o AuNPs is interpolated to be 5x higher at 0.73 µJ. Thus, vesicles with AuNPs present in the membrane displayed significantly greater photosensitivity under fs irradiation.

Under ns-irradiation, the difference in energy to bring about similar rupture percentages was even greater than the case of fs-irradiation, as shown in Figure 3D. Vesicles without AuNPs experienced a rupture rate of 100% at 15 µJ, which decreases to 39% at 9.3 µJ, and by 3.7 µJ, rupture was no longer observed. Vesicles with AuNPs show a distinct reduction in energy to bring about rupture. Even at the lowest pulse energy of 0.37 µJ, polymersomes containing plasmonic nanoparticles still experience rupture at a frequency of 17%. This represents an interpolated 14-fold decrease in the rupture threshold for polymersomes containing AuNPs under ns-irradiation. Moreover, at 9.3 µJ, vesicles with AuNPs ruptured 100% of the time in comparison to only 38% for those without.

### 3.2. Using Pulse Duration for Mechanistic Insight

The results above clearly suggest enhanced photosensitivity of vesicles containing plasmonic nanoparticles in the membrane in response to both fs and ns pulses. However, the vast difference in required pulse energies prompts questioning from a mechanistic standpoint given the differences in pulse durations, i.e., 100 fs vs. 7 ns. After excitation of the AuNPs within the membrane, energy must escape the excited AuNPs; the mechanism by which this occurs will have an effect on the self-assembly of the copolymer and ultimately determine the threshold for rupture. Primarily thermal mechanisms will allow for heat to diffuse away from the particle and into the surrounding copolymer, thus altering self-assembly. Alternatively, more energetic effects such as the formation of a rapidly expanding vapor bubble around the particle, or fragmentation of the AuNP by coulombic explosion can occur, leading to membrane disruption. The reduction in energy between fs and ns plasmonic excitation for 2.5 nm AuNPs can be explained, at least from a thermal standpoint, by a recent article published by Metwally et al. [25]. In their article, they investigated the fluence required for the water immediately outside of variously-sized AuNPs to reach the spinodal point which initiates formation of a vapor layer around the particle. They looked at both ns and fs pulse durations with a wavelength 532 nm. Results showed that, for sub-20 nm AuNPs, the fluence needed to reach the spinodal temperature was significantly larger for nanosecond excitation than with femtosecond irradiation. Thus, it is proposed that the thermal dissipation of energy out of the particles during delivery of a ns pulse suppresses the peak temperature of the water layer. Femtosecond irradiation does not experience this thermal-dissipation during the pulse duration because all of the incident energy is delivered to the particle in a time frame less than the electron–phonon (~1 ps) and phonon–phonon (~100 ps) relaxation times [26]. Hence, the corresponding thermal energy is delivered essentially at once to the water surrounding the particle and a higher temperature can be attained for a given fluence. In the case of AuNPs loaded in the membrane of polymersomes, reaching the spinodal temperature of water may not be critical, given that PBD has a relatively low water permeability, however it is reasonable to conclude that some critical temperature exists that initiates membrane disruption such that rupture results, especially in the case of ns irradiation. Thus, it is expected that nanoparticle energy dissipation will be attributed to different primary processes within the two regimes.

To further probe the mechanistic differences in rupture initiation, the fate of the nanoparticles in the membrane both before and after ns and fs irradiation were investigated using high-resolution transmission electron microscopy (HR-TEM). The pulse durations used, 100 fs and 7 ns, are of particular interest as they lie on either side of the electron phonon coupling time [27]. While micron-scale vesicles provide an excellent size regime to facilitate single vesicle imaging, polymersomes on the nano-scale are better suited to examine the fate of the nanoparticles in response to excitation because all loaded particles within a vesicle will be subject to irradiation due to the smaller size of the vesicle in comparison to the beam profile. Stirring of the colloidal solution ensures exposure of the entire population of vesicles during continuous pulsed irradiation.

**Figure 4:**
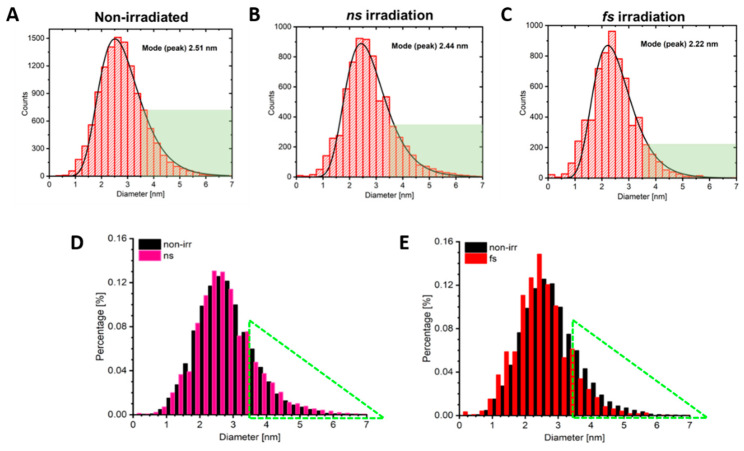
AuNP size distributions following vesicle irradiation. (**A**–**C**) The diameters of AuNP populations within polymersome membranes were measured without irradiation, and after ns and fs irradiation, respectively. The green shaded area highlights NPs with diameters greater than 3.5 nm. (**D**,**E**) Comparative population overlays for irradiated vs. non-irradiated samples. The green triangle highlights the size region of interest where particle fragmentation is apparent.

The AuNP size distribution along with the mode for each sample is shown in Figure 4 (with corresponding representative HR-TEM images included in Figure S4). In summary, the mode peak size for the non-irradiated, ns-irradiated, and fs-irradiated samples are 2.51 nm, 2.44 nm, 2.22 nm, respectively. While the AuNPs are trending towards smaller diameters with decreasing pulse duration, the difference becomes more apparent upon the analysis of the population of AuNPs greater than 3.5 nm. For each sample, this region is represented by the green shaded area in Figure 4A–C. For the non-irradiated polymersome sample and the ns-irradiated polymersome sample, the percentage of AuNPs greater than 3.5 nm is approximately 13%, whereas this region only represents 7% of the total population for the fs-irradiated polymersome sample. This suggests that a larger population of nanoparticles undergo size reduction in response to fs irradiation which is consistent with findings by Delfour et al., in that larger particles will preferentially undergo fragmentation under fs irradiation [28].

Figure 4D,E shows the data overlay of the irradiated and non-irradiated samples for both ns and fs pulse durations, respectively. Both irradiated distributions are significantly different from the non-irritated particles at a level of 0.05 using a parametric z-test and log transformed data. The green triangle highlights the size region of interest where particle fragmentation is apparent. Concurrently, the opposite trend is seen for AuNPs within a smaller size range. The amount of AuNPs which fall below 2.5 nm trend towards larger counts for the fs-irradiated sample in comparison to the control sample, whereas this trend is not visible for ns irradiation. This suggests that pulse duration plays a significant role in vesicle rupture mechanism. In the case of ns irradiation, thermal relaxation through electron–phonon coupling begins to occur before the pulse delivery has been completed. Thus, thermal energy dissipation predominates and minimal fragmentation will occur. However, in the case of fs irradiation, the entirety of the pulse is delivered before energy can be dissipated through thermal pathways. Thus, this high energy density results in nanoparticle fragmentation.

### 3.3. Controlling Complete Rupture vs. Pore Formation with Decreased Pulse Energy

**Figure 5:**
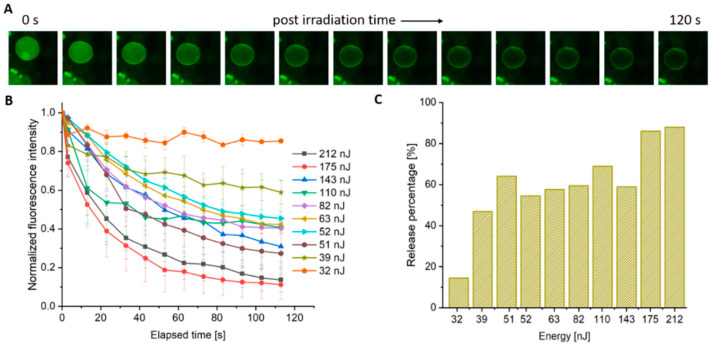
Encapsulant release as a function of time from Polymersomes loaded with 0.04% (*w/v*) AuNPs. (**A**) A sample time series displaying a decrease in fluorescence intensity as a function of time as FITC-dextran diffuses out of the vesicle after the delivery of a single 212 nJ pulse. (**B**) The fluorescence intensity inside of an individual vesicle is measured as a function of time following a single 532 nm, fs pulse at the indicated energy. Each line represents the fluorescence decay in a region of interest (ROI) (3–4 replicates +/− standard error of the mean (SEM)). (**C**) Total encapsulant release after 120 s at the indicated pulse energy.

The rupture frequency plots in Figure 3 show that irradiation below the rupture threshold does not result in complete rupture and, hence, the vesicles remain visibly intact via fluorescence microscopy. To probe membrane integrity and permeability at sub threshold levels, the polymersome cores were loaded with a hydrophilic fluorescent dye (FITC-dextran). It was observed that irradiation at these lower pulse energies leads to the release of the aqueous contents over a finite timescale as opposed to the instantaneous release event experienced with rupture. This behavior suggests that the membrane has undergone a poration event. At pulse energies with a low likelihood of rupture, the overall spherical shape of the vesicle can remain intact with little to no perceivable buckling and/or distortion of the membrane. Figure 5A shows a time series of fluorescent images of such an event.

In order to examine release characteristics of the porated vesicles, a series of fluorescent images were taken at regular intervals (10 s) that began approximately 3 s after the arrival of the incident laser pulse. The temporal release of encapsulant was determined by selecting a region of interest (ROI) both within the targeted polymersome and in the background (to account for possible photobleaching due to imaging post-irradiation) and then normalizing that value to the pre-irradiated intensity (see example calculation in Table S1). Results for fs irradiation are shown in Figure 5B where a minimum of three (non-rupturing) vesicles were measured at each pulse energy and graphed as the mean, with error bars denoting the standard error of the mean (SEM).

Given that a rupture frequency of approximately 50% is observed for pulse energies at 210 nJ, this served as the upper energy boundary for the poration study. As shown in Figure 5B, the fluorescence intensity decreases in an exponential manner for all energies except at the lower boundary of 32 nJ where the membrane presumably reseals on a timescale equal to or shorter than the initial time step (i.e., 3 s). For the case of pulse energies near the upper boundary (140–220 nJ), encapsulant release occurs rapidly with a large degree (>80%) of the vesicle contents escaping (Figure 5C). Energies in the middle of the range (50–110 nJ) result in steady release with the removal of 50–60% of the contents. Given that the fluorescence continues to diminish up to the endpoint, it can be inferred that these pores are stable (i.e., did not grow boundless to rupture, or close) during the 120 s timeframe. Irradiation with pulse energies at the lower boundary (32 nJ) leads to a small reduction (13%) in the fluorescence signal which then quickly levels-off, indicating a short-lived pore that quickly self-heals. Note, attempts to observe temporal release under ns irradiation were erratic which indicates the formation of unstable pores, and hence were not included.

To confirm that plasmonic excitation of the AuNP was playing an important role in pore formation, a control experiment was performed with polymersomes assembled without nanoparticles. While poration can still be observed in these empty membrane vesicles, a much higher pulse energy is required (Figure S5). Exposure of polymersomes without AuNPs to a 532 nm, 200 nJ, fs pulse resulted in <20% total encapsulant release. Compared to a membrane with AuNPs, the release percentage is near 90% with a rupture frequency close to 50%.

An interesting discrepancy was observed between energy threshold values for rupture and poration studies when hydrophilic encapsulant, FITC-dextran, was incorporated into the aqueous lumen. There is some precedent in the literature that the encapsulation of varying concentrations and molecular weights of dextrans into the aqueous lumen of photoresponsive polymersomes can alter vesicle deformation frequency [16]. We investigated the extent of the impact of this interaction on our rupture study by evaluating micron-sized PBD_35_-*b*-PEG_20_ polymersomes formed both with and without FITC-dextran in the aqueous core. Rupture thresholds were determined in response to an fs pulse for samples containing and excluding AuNPs. When juxtaposed to Figure 3, Figure S6 shows that both vesicles prepared with and without AuNPs are observed to undergo complete rupture at a lower pulse energy when they do not contain FITC-dextran in their aqueous lumen. This result of decreased photosensitivity with dextran encapsulation is opposite to the trend found by Kamat et al., demonstrating an increase in photosensitivity with dextran incorporation. The authors observed an increase in deformation frequency and a decrease in elastic modulus, for two different PBD-*b*-PEG diblock copolymers, that scaled with the dextran molecular weight. The mechanism put forth states that the dextran penetrates the hydrophilic corona and reduces interfacial tension, thereby increasing the area of the inner leaflet. This difference in surface area can result in asymmetrical stress in the bilayer and destabilize the membrane. However, this reported effect was weaker for lower molecular weight dextrans, such as that conjugated to the FITC dye in this study (3–5000 Da). Several important distinctions between this work and that in the Kamat study are: (1) the inclusion of AuNPs in the membrane, (2) the molecular weight of the dextran used and (3) the use of ultrafast irradiation instead of CW. Further investigation is warranted to clarify the role played by dextrans in various photoresponsive polymersome systems.

### 3.4. Role of AuNP Concentration on Vesicle Poration

**Figure 6:**
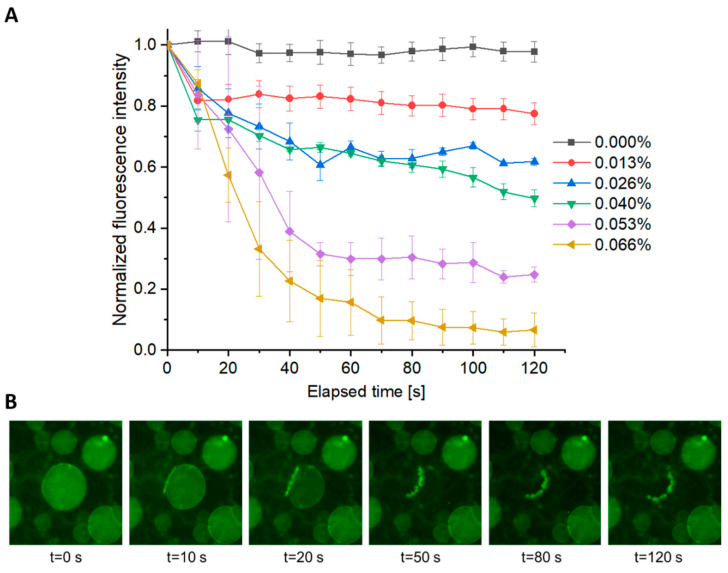
Effect of AuNP concentration on encapsulant release. (**A**) The fluorescence intensity inside of an individual vesicle is measured as a function of time following a single 532 nm, fs pulse (144 nJ). Each line represents the fluorescence decay in an ROI (3–4 replicates +/− SEM) with varying concentrations of AuNPs encapsulated within the polymersome membrane. (**B**) Poration leading to structural instability. A polymersome loaded with 0.066% (*w/v*) AuNPs is subjected to a single fs pulse (144 nJ). Upon irradiation, a large pore can be observed. The vesicle remains intact for 10–20 s following the pulse until the vesicle structure is compromised after approximately 50 s.

To investigate the effect of AuNP concentration on polymersome photo-response, vesicles with varying amounts of AuNPs loaded in the membrane were prepared. Polymersome samples with no AuNPs, 0.013%, 0.026%, 0.040%, 0.053%, and 0.066% (*w/v*) AuNPs were subjected to a single, 144 nJ fs pulse (chosen as a mid-range poration energy) and the subsequent cargo diffusion rate and total release were quantified for each sample. A distinct difference in the amount of fluorophore released from a minimally loaded sample with 0.013% AuNPs compared to a maximally loaded sample with 0.066% AuNPs could be observed, and is displayed in Figure 6A. In the case of the minimally loaded sample, there is negligible change in the fluorescence intensity in the vesicle core. However, in the case of the maximally loaded sample, a significant amount of the fluorophore diffused from the vesicle after two minutes. Using identical methods as those used to acquire data in Figure 5B, Figure 6A displays normalized fluorescence intensity as a function of time for a minimum of three vesicles at six distinct AuNP concentrations. Encapsulant release ranges from 90–100% (in the case of 0.066% (*w/v*) AuNPs) to 0% (in the case of 0% *w/v* AuNPs), scaling with AuNP concentrations. 

Interestingly, in a small population of maximally loaded vesicles (0.066% *w/v* AuNPs), structural changes resulting in vesicle destabilization were observed. This occurrence is distinctly different from the case of complete rupture which occurs nearly instantaneously upon pulse delivery. As shown in Figure 6B, the vesicle is destabilized as a function of time concurrent with cargo diffusion. This structural instability mostly occurs when the ratio of pore to vesicle size reaches a critical value at which the pore is unable to remain stable or self-heal, resulting in complete rupture.

## 4. Discussion

The ability to transport molecules in an unreacted state has significant importance for a variety of applications, namely, microreactors and drug delivery. Polymersomes have proven to be a promising candidate for such applications due to their robust nature and tunability. The self-assembly of PEO_20_-*b*-PBD_35_ yields vesicles which are inherently are unreactive to stimuli, but in the work presented here, the incorporation of nanoparticles into the hydrophobic region of the membrane has proven to efficiently photosensitize the system in response to short pulse irradiation. The threshold energy for complete rupture was shown to reduce by a factor of five under fs irradiation which increased to a factor of 14 for ns irradiation.

By changing the pulse energy, cargo release can be precisely controlled ranging from instantaneous delivery, i.e., complete rupture, to regulated release over a timescale from seconds to minutes through pore formation. Additionally, the concentration of AuNPs loaded in the hydrophobic region of the membrane plays a role in cargo diffusion rates. This is significant for future biological applications where limiting the amount of AuNPs in the system may be favored.

The likely mechanism for membrane disruption is nanobubble formation around the excited nanoparticles upon thermal relaxation. In the case of ns irradiation, there is sufficient time for thermal transport which maximizes bubble formation. Such bubbles disrupt overall membrane stability resulting in complete rupture. This is indicated by the erratic nature of pore formation under ns irradiation. As evidenced by the size distributions of pre- and post-irradiated nanoparticles that have been incorporated into the membrane, those subjected to ns irradiation did not show signs of particle fragmentation while indications of size reduction are present in response to fs irradiation. The process of fragmentation decreases the localized thermal energy available for nanobubble formation, thus resulting in smaller regions of disruption and more stable pores.

This fundamental study has the potential to influence future work in the field of microreactors and drug delivery, where spatiotemporal control over cargo release is of central importance; however, implementation of the system in drug delivery applications will require scaling down to the more biologically appropriate nano-regime. Additionally, anisotropic gold nanoparticles may prove useful in shifting the response wavelength to the near-IR region of the spectrum for deeper tissue penetration in vivo. Work is currently underway to translate these findings from the micron- to the nano-regime.

## Supplementary Materials

The following are available online at https://www.mdpi.com/2073-4360/12/10/2381/s1, Figure S1: Gel-assisted rehydration method for polymersome preparation, Figure S2: Ultrafast (fs) laser-microscope setup, Figure S3: Spot size determination, Figure S4: Representative HR-TEM images of nano-polymersomes containing AuNPs after irradiation, Figure S5: Release profile of encapsulant from polymersomes without AuNPs, Figure S6: Rupture threshold for vesicles without FITC-dextran in the core, Table S1: Example calculation for normalized intensity as shown in Figure 5, Figure 6 and Figure S5.
