# Supplementary material for: Polymersome Poration and Rupture Mediated by Plasmonic Nanoparticles in Response to Single-Pulse Irradiation
Source: Polymers (Basel). 2020 Oct 16;12(10):2381. doi: 10.3390/polym12102381 (PMC7602809; doi:10.3390/polym12102381)
Supplement: Supplementary file 1 [file polymers-12-02381-s001.pdf]

polymers

MDPI

Article

Polymersome Poration and Rupture Mediated by Plasmonic Nanoparticles in Response to Single-Pulse Irradiation

Gina M. DiSalvo [1,†], Abby R. Robinson [1,†], Mohamed S. Aly [2], Eric R. Hoglund [3], Sean M. O'Malley [2,4] and Julianne C. Griepenburg [2,4,*]

[1] Department of Chemistry, Rutgers University-Camden, 315 Penn Street, Camden, NJ 08102, USA; gdisalvo22@gmail.com (G.M.D.); robinsonabby13@gmail.com (A.R.R.)
[2] Department of Physics, Rutgers University-Camden, 227 Penn Street, Camden, NJ 08102, USA; msa194@scarletmail.rutgers.edu (M.S.A.); omallese@camden.rutgers.edu (S.M.O.)
[3] Department of Materials Science and Engineering, University of Virginia, Thornton Hall, P.O. Box 400259, Charlottesville, VA 22904, USA; erh3cq@virginia.edu
[4] Center for Computational and Integrative Biology, Rutgers University-Camden, Camden, NJ 08102, USA
* Correspondence: j.griepenburg@rutgers.edu; Tel.: +1-856-225-6293
† Denotes equal contribution.

Received: 26 September 2020; Accepted: 13 October 2020; Published: 16 October 2020

Supporting Information

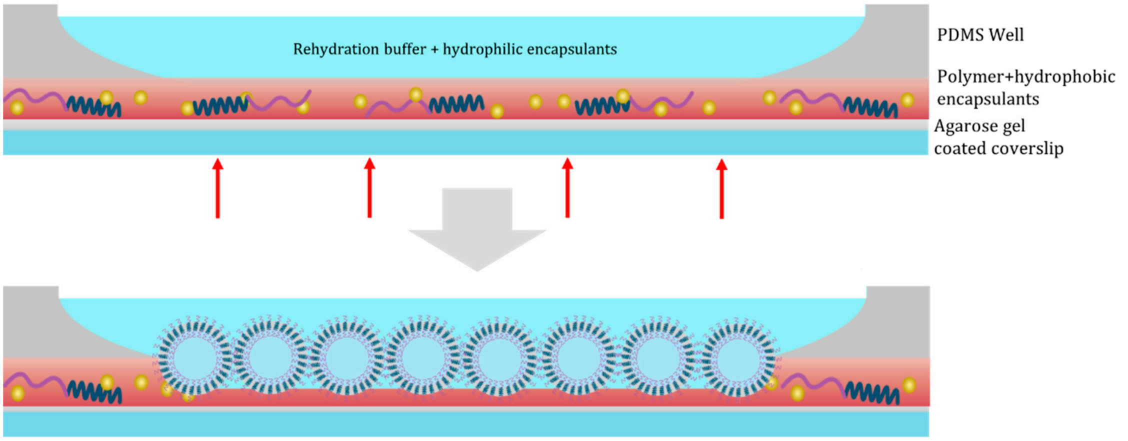

Figure S1. Gel-assisted rehydration method for polymersome preparation.

Schematic representation of polymersome preparation via the gel-assisted rehydration method. Upon addition of rehydration buffer and heat to the sample, the copolymer film begins to swell and forms vesicles atop of the agarose film. The micron-sized polymersomes remain partially attached to the surface facilitating single-vesicle studies.

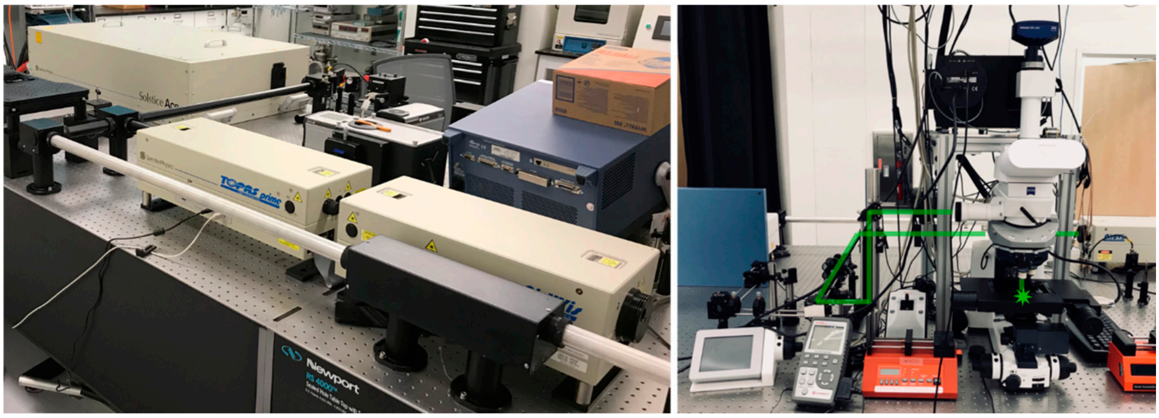

Figure S2. Ultrafast (fs) laser-microscope setup.

A Ti:Sapphire laser system (shown) or Nd-YAG ns laser (not shown) is steered and focused into the Zeiss Axio Examiner upright microscope for irradiation and imaging studies. The beam path is depicted by the green line.

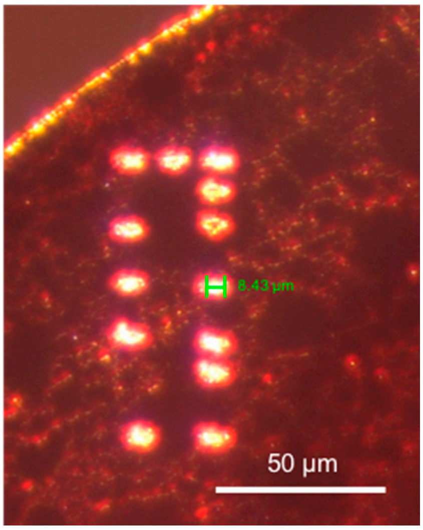

Figure S3. Spot size determination.

Spot size is determined using a glass bottom petri dish covered with a series of ink blots. Water is added to the dish to mimic the aqueous experimental conditions. The ink is ablated at several spots, each by a single pulse, and the resulting damage spots are measured which serves as a gauge of the irradiated area. Note, damage spot is reported instead of beam waist, due to difficulties in measuring beam waists associated with the short focal lengths and aqueous environments for which these studies take place. Hence, irradiations are reported in terms of energy rather than fluence. Damage spot sizes were kept constant ranging from 8-9 µm.

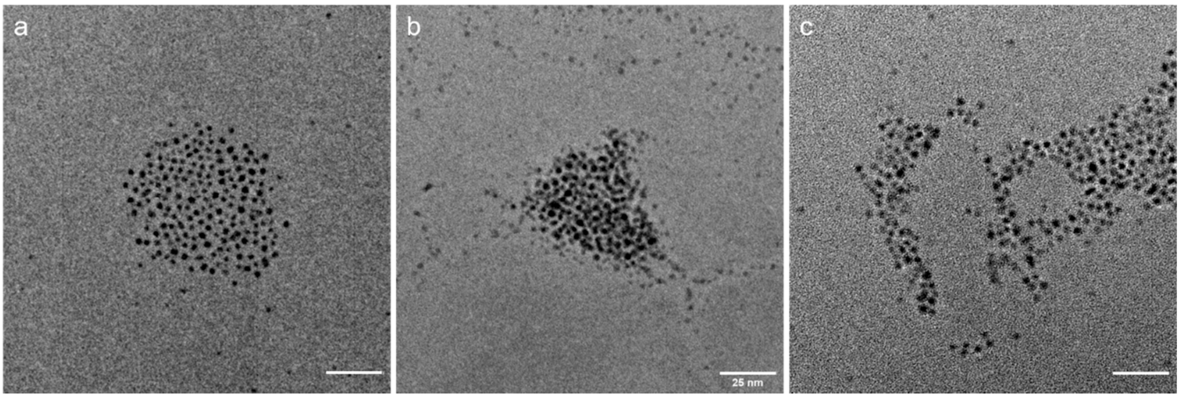

Figure S4. Representative HR-TEM image of nano-polymersomes containing AuNPs after (a) nanosecond irradiation, (b) femtosecond irradiation, and (c) no irradiation. All scale bars are 25 nm.

Images were taken at 69,000x magnification and scale bars represent 25 nm. The nanoparticles, on the TEM grids, were often not found in circular groupings likely due to the drying process leading to vesicle rupture. Multiple images were taken for each sample and particle sizes were analyzed for a minimum of 5,000 particles per sample.

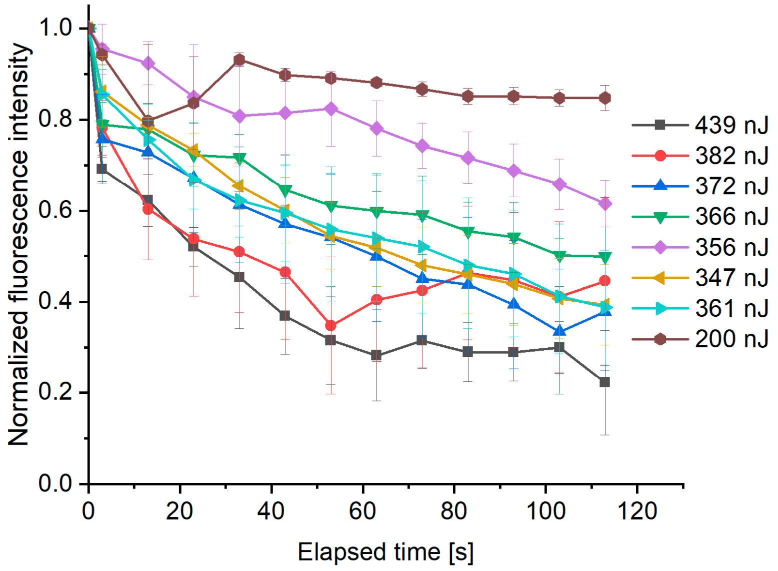

Figure S5. Release profile of encapsulant from polymersomes without AuNPs.

Normalized fluorescence intensity of FITC-dextran inside the core of polymersomes without AuNPs in the membrane. Vesicles were individually irradiated with a single, 532 nm fs pulse at the indicated pulse energies. Data is shown as the mean of at least three vesicles and error is reported as SEM.

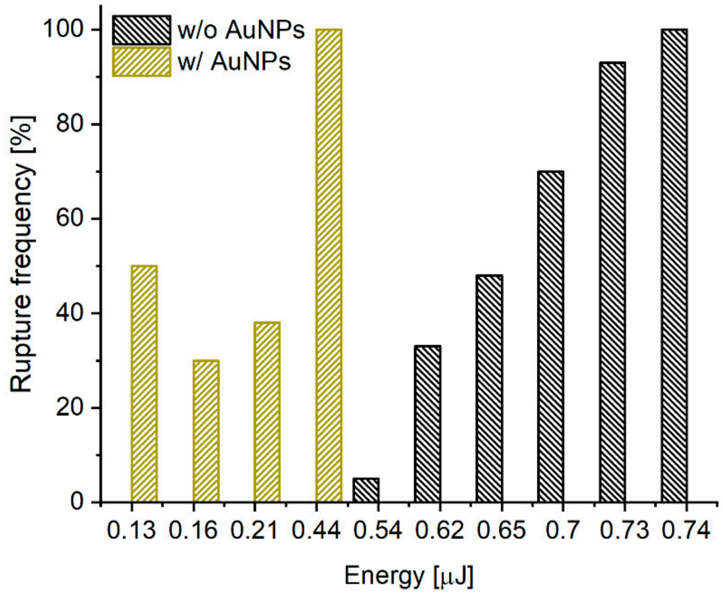

Figure S6. Rupture threshold for vesicles without FITC-dextran in the core.

Each bar represents the percentage of polymersomes, either with or without AuNP loading, that ruptured completely in response to a single, 532 nm, fs pulse at the specified energy.

TIME (SEC)	INTENSITY	BACKGROUND	CORRECTED INTENSITY	NORMALIZED INTENSITY
0	246.012	99.138	146.874	1.000
3	217.200	98.719	118.481	0.807
13	178.771	100.386	78.385	0.534
23	156.263	99.510	56.753	0.386
33	143.353	99.279	44.074	0.300
43	137.145	98.605	38.540	0.262
53	133.314	98.826	34.488	0.235
63	132.009	99.534	32.475	0.221
73	129.789	98.084	31.705	0.216
83	127.915	98.950	28.965	0.197
93	126.216	98.873	27.343	0.186
103	123.239	97.254	25.985	0.177
113	123.043	97.137	25.906	0.176

Table S1: Example data for normalized intensity as shown in Figures 5, 6, and S5.

Release curve data for polymersomes with AuNP loading irradiated at 212 nJ. The fluorescence intensity was determined in the selected ROI and was corrected by subtracting the intensity of an ROI set in the background. The ROIs were placed in the same position for every image within a time series.

Publisher's Note: MDPI stays neutral with regard to jurisdictional claims in published maps and institutional affiliations

© 2020 by the authors. Submitted for possible open access publication under the terms and conditions of the Creative Commons Attribution (CC BY) license (http://creativecommons.org/licenses/by/4.0/).
